# Magnetic resonance imaging of oxygen microbubbles

**DOI:** 10.1049/htl.2018.5058

**Published:** 2019-08-21

**Authors:** Elinor Thompson, Sean Smart, Paul Kinchesh, Daniel Bulte, Eleanor Stride

**Affiliations:** 1 Institute of Biomedical Engineering, Department of Engineering Science, University of Oxford, OX3 7DQ, UK; 2 Radiobiology Research Institute, Department of Oncology, University of Oxford, OX3 7DQ, UK

**Keywords:** phantoms, hydrogels, biomedical MRI, cancer, patient treatment, bubbles, tumours, biomedical ultrasonics, oxygen, magnetic resonance imaging, oxygen loaded microbubbles, tissue oxygenation, T1, longitudinal relaxation time measurements, microbubble concentration, tumour hypoxia, cancer therapy, hydrogel phantoms, ultrasound application, physiological conditions, MRI

## Abstract

Oxygen loaded microbubbles are being investigated as a means of reducing tumour hypoxia in order to improve response to cancer therapy. To optimise this approach, it is desirable to be able to measure changes in tissue oxygenation in real-time during treatment. In this study, the feasibility of using magnetic resonance imaging (MRI) for this purpose was investigated. Longitudinal relaxation time (T1) measurements were made in simple hydrogel phantoms containing two different concentrations of oxygen microbubbles. T1 was found to be unaffected by the presence of oxygen microbubbles at either concentration. Upon application of ultrasound to destroy the microbubbles, however, a statistically significant reduction in T1 was seen for the higher microbubble concentration. Further work is needed to assess the influence of physiological conditions upon the measurements, but these preliminary results suggest that MRI could provide a method for quantifying the changes in tissue oxygenation produced by microbubbles during therapy.

## 1 Introduction

Solid cancerous tumours frequently contain regions in which the partial pressure of oxygen drops significantly below that of neighbouring healthy tissue. This oxygen deficiency is described as hypoxia. In cancerous tumours, hypoxia results from the demand for oxygen from rapidly proliferating cancer cells being higher than that available from the blood supplied by the irregular tumour vasculature [1]. Hypoxia reduces the efficacy of multiple forms of cancer treatment. This is primarily because radiotherapy and many types of chemotherapies rely on the generation of cytotoxic oxygen-free radicals, which cannot be generated in sufficient quantities in hypoxic tissue [2]. Furthermore, the adaptations induced in cells by hypoxic environments promote cell mobility and survival, which lead to an increased likelihood of metastasis and therefore reduce the effectiveness of surgery [1]. Oxygen loaded microbubbles are among a number of methods being developed to deliver oxygen to hypoxic tumours to improve the response to therapy. Once they have been injected, ultrasound can be used to release the oxygen at the tumour site via acoustic cavitation [3–6].

Surfactant stabilised microbubbles with a diameter between 1 and 10 μm have been widely used as ultrasound contrast agents since the 1990s, but more recently their use in drug delivery, gene therapy, and ablative therapies has also been investigated [7, 8]. Several studies have demonstrated that oxygen loaded microbubbles can be used for extra-pulmonary ventilation [5]. Their impact on cancer treatment has also been investigated in vitro and in vivo for radiotherapy [3, 4] and combined sonodynamic and chemotherapy [5, 6]. In these studies, the bubbles are injected intratumourally [5, 6] or intraperitoneally [4], and low-intensity ultrasound is used to destroy the bubbles, releasing oxygen at the tumour site (as well as activating the drug in the case of sonodynamic therapy). The results have indicated considerable improvements in tumour response. As this approach undergoes further development, there is a need for a robust, non-invasive imaging technique to monitor the lifetime of the microbubbles in vivo and the oxygenation at the tumour site. Whilst the former can be achieved using ultrasound contrast imaging, clinically relevant techniques for the latter are lacking. The aim of this project is to investigate the feasibility of magnetic resonance imaging (MRI) to address this need.

Molecular oxygen is paramagnetic, so its presence in solution generates fluctuating magnetic fields that promote the longitudinal relaxation of protons in nearby water molecules. This effect is known as paramagnetic relaxation enhancement [9]. In MRI, this translates into a reduction in longitudinal relaxation time (T1) with increased oxygen concentration. There has been shown to be a linear relationship between oxygen partial pressure (pO_2_) and 1/T1 in pure solutions [10]. Although these effects are relatively small in vivo, recent developments in hardware and analysis have made measuring changes in T1 a viable method for monitoring tissue oxygenation [11, 12], and have been shown to be an effective way to measure tumour hypoxia [13–15]. In contrast to the standard method of measuring pO_2_ with oxygen electrodes, T1 mapping is non-invasive, combines quantitative measurements with imaging, and can be used on a tumour site anywhere in the body. Unlike positron emission tomography, another modality for imaging hypoxia, MRI does not require exposing patients to ionising radiation. In addition, proton MRI is already widely available in hospitals so it would be straightforward to translate this method into the clinic.

The primary mechanism of paramagnetic relaxation enhancement is the dipole–dipole interaction between the hydrogen atoms in the water molecules and oxygen. The strength of this interaction is inversely proportional to the sixth power of the distance between the molecules [16]. Therefore oxygen enclosed in a bubble would not be expected to produce a significant T1 reduction. With this distinction in mind, it was proposed that MRI could be used to gain a better understanding of microbubble lifetime and oxygen release mechanisms, as oxygen enclosed within a bubble would not cause a reduction in T1 until the bubble is ruptured and the gas is released into solution. Previous work with microbubbles and MRI has focused on changes in transverse relaxation (T2 and T2*) arising from the magnetic susceptibility effects of the gas–liquid interface [17–19]. To the best of the authors’ knowledge, this is the first investigation of the effect on MRI of oxygen loaded microbubbles.

## 2 Materials and methods

### 2.1 Materials

1,2-dibehenoyl-*sn*-glycero-3-phosphocholine (DBPC) was purchased from Avanti Polar Lipids (Alabaster, AL, USA). Polyethylene glycol (PEG)-40 stearate, chloroform, Dulbecco's phosphate-buffered saline (PBS), glycyrrhizic acid and glycerol were purchased from Sigma-Aldrich Ltd (Gillingham, Dorset, UK). Oxygen, nitrogen and sulphur hexafluoride (SF6) cylinders were purchased from BOC gases (Guildford, Surrey, UK). Ultrasound gel was purchased from Ana Wiz Ltd (Surbiton, Surrey, UK).

### 2.2 Microbubble preparation

Following the protocol developed in [20] DBPC and PEG-40 stearate were dissolved in chloroform and placed in a glass vial in a molar ratio of 9:1, using 15.98 and 4.02 mg, respectively. The solution was heated at 50°C for at least 12 h until all the chloroform had evaporated. 5 ml of PBS was added to the lipid film and the solution was magnetically stirred at 100°C for 1 h to resuspend the constituents. The suspension was then sonicated using a probe sonicator (QSonica Q125, 20 kHz, 3 mm probe tip) for 90 s at a root-mean-square (RMS) power of 4 W and placed in a hot water bath at a temperature of ∼85°C. After 5 min, the sample was removed from the water bath and sonicated at RMS power of 4 W for a further 20 s. The headspace of the vial was then filled with SF6 gas and the gas–liquid interface sonicated for 20 s at RMS power of 24 W to produce the microbubbles. The microbubble suspension was cooled in an ice bath for ∼10 min. To replace the SF6 gas core with oxygen, the solution was exposed to a steady flow of oxygen gas (∼54 mbar) for 2 min, and then resealed and placed in an ice bath for a further 5 min. Nitrogen microbubbles were generated in the same way as oxygen microbubbles, with nitrogen gas used instead of oxygen.

### 2.3 Characterisation

Microbubble samples were imaged using optical microscopy to determine their size distribution and concentration and to monitor any degradation in the gel phantoms over the time scales relevant to subsequent experiments. 1 ml samples were removed from the microbubble suspensions and diluted with 5 ml water or 2.5 ml water and 2.42 ml ultrasound gel. At 15–20 min intervals, 1 ml aliquots from the gel-microbubble solution were then diluted further with 1 ml PBS before they were imaged. Between 20 and 30 images were obtained of each sample using a 40× objective lens with a Leica DM500 optical microscope. The microbubble size distribution and concentration were then obtained using purpose written image analysis software in Matlab (2017a, The MathWorks, Natick, MA, USA). This procedure was repeated twice on different days.

A CDI blood parameter monitoring system (Terumo UK Ltd, Egham, Surrey) was used to measure the oxygen partial pressure of the microbubble solutions before and after the application of ultrasound. All tests were carried out in a closed loop with a peristaltic pump (Gilson, Minipuls3, Cole Parmer, Hanwell, UK) operating at a flow rate of 16 ml/min and a length of tubing into which the sensor was inserted. A gel–O_2_ microbubble mixture (1.1 ml ultrasound gel, 1.2 ml filtered deionised water, 0.92 ml O_2_ microbubbles) was used. Measurements were recorded at 10 s intervals over 2 min, then for 1 min while the tubing was submerged in an ultrasound bath operating at a power of 150 W (Eumax Corp., New Taipei City, China), and then for a further 10 min. Additional readings were obtained for a total of 13 min with no ultrasound applied. The process was repeated with oxygen saturated water in the flow loop.

### 2.4 Magnetic resonance imaging (MRI)

All studies were performed using a 7.0 tesla 210 mm horizontal bore VNMRS preclinical imaging system equipped with 120 mm bore gradient insert (Varian Inc., Palo Alto, CA, USA). The gradient insert specification was: maximum gradient strength 400 mT/m in all axes; and duty cycle 14% at maximum gradient strength, simultaneously in all three axes. Radiofrequency (RF) transmission and reception were performed with a 30 mm long, 25 mm ID quadrature birdcage coil (Rapid Biomedical GmbH, Germany). For this project, variable flip angle (VFA) imaging was used to generate T1 maps because it provides accurate 3D T1 mapping at a fraction of the time required for the standard inversion recovery method [21].

All VFA scans were preceded by an actual flip-angle imaging (AFI) scan [22], in order to provide an accurate spatial map of B1 within the RF coil. A 3D gradient echo sequence was used with repetition time (TR) TR_1_ = 10 ms, TR_2_ = 100 ms, echo time (TE) = 0.59 ms, field of view (FOV) = 54 × 27 × 27 mm^3^, matrix = 64 × 32 × 32, RF spoiling with a phase increment of 117° [23], gradient spoiling with 300 mT/m for 5.4 ms in all three axes, RF hard pulse duration 128 μs and FA = 56°. AFI data were Hanning filtered and zero filled to matrix size 128 × 64 × 64 to provide a voxel-wise correction for the FAs prescribed in VFA during T1 analysis.

VFA imaging was performed with a 3D gradient echo sequence with TR = 2.8 ms, TE = 1.0 ms, FOV = 54 × 27 × 27 mm^3^, matrix = 128 × 64 × 64, RF spoiling with a phase increment of 117°, gradient spoiling with 128 mT/m for 1.0 ms in all three axes, RF hard pulse duration 16 μs, and 16 FAs nominally set from 1° to 7° in steps of 0.4°. The non-linear least squares T1 determination from the 3D data typically took 6 s. Approximately five iterations were required for minimisation with 16 different FAs. Circular regions of interest were then drawn within the sample tubes on a T1 map taken across a cross section of the tubes to generate T1 statistics (mean, median, standard deviation) for each one.

#### 2.4.1 MRI experiments

In all experiments, the samples were loaded into 0.5 ml syringes, which were then sealed with wax. Care was taken to remove any large gas bubbles from the sample tubes before they were sealed. Samples were then placed into a custom-built holder to ensure consistent positioning relative to the scanner. Five vials were prepared for the first experiment, each containing 2.42 ml ultrasound gel and 2.5 ml water. Gel was used instead of water to reduce the effects of buoyancy and ensure a homogeneous suspension of microbubble throughout the duration of the scan. 1 and 0.25 ml aliquots of O_2_ microbubble and N_2_ microbubble suspensions were added separately into four of the vials and mixed until the solutions were fully dispersed. All samples were made up to total a volume of 5.92 ml with PBS. Two further gel phantoms were prepared (2.42 ml gel, 2.5 ml water, 2 ml PBS), and sparged with oxygen for 20 and 120 s to provide two differing oxygen concentrations. T1 maps were acquired of all the above samples. Significance between the T1 values measured with VFA in the microbubble samples relative to the control was assessed using a two-tailed Student's *t*-test, with a Bonferroni correction for multiple comparisons.

For the control samples containing no microbubbles, a gel mixture was prepared (14.52 ml gel, 15 ml water, 6 ml PBS) in a beaker and degassed under vacuum. The mixture was separated equally into three vials. The first vial was sparged with oxygen gas and samples were extracted for MRI scanning after 8, 16, 24 and 120 s. Samples were extracted from the second vial after sparging with nitrogen gas for 8 and 24 s. One sample was taken from the third vial, and a pO_2_ reading was taken from the remaining sample with a dissolved oxygen dip-probe (Cole-Parmer Instrument Company, London, UK). The samples were scanned with the VFA protocol outlined above.

To investigate the effect of ultrasound exposure, the following mixtures were prepared: 4.82 ml ultrasound gel + 1 ml O_2_ microbubble suspension; 4.82 ml ultrasound gel + 2 ml O_2_ microbubble suspension; 4.82 ml ultrasound gel + 1 ml N_2_ microbubble suspension; 4.82 ml ultrasound gel + 2 ml N_2_ microbubble suspension; 4.82 ml ultrasound gel only. All vials were made up to a total volume of 6.82 ml with PBS. Two samples were taken for scanning from each of the O_2_ microbubble vials, and one from each of the rest. The samples were scanned with 12 consecutive AFI–VFA sequences for a total of 63 min. Microbubble samples with the same formulations as above were then scanned before and after being exposed to ultrasound. After the first VFA scan, the syringes were placed in an ultrasound bath with a RMS power of 60 W (Grant Instruments, Cambridge, UK) for 10 s. The plungers were then depressed and held in place with the syringe caps to avoid the formation of a gas pocket at the top of the syringe during the second scan. The significance of the change in T1 between scans was assessed using a two-tailed, paired Student's *t*-test.

## 3 Results and discussion

### 3.1 Characterisation

#### 3.1.1 Microscopy

Photomicrographs were obtained indicating that the mean average diameter of the O_2_ microbubbles was ∼2 μm with a polydispersity index of 20% and the concentration was 3 × 10^8^ microbubbles/ml. Samples were also extracted from the gel mixture and analysed over a period of 90 min. No significant change was seen in the microbubble concentration over this period, which was the maximum time between making the samples and acquiring the MRI data.

#### 3.1.2 pO_2_ flow loop measurements

**Fig. 1:**
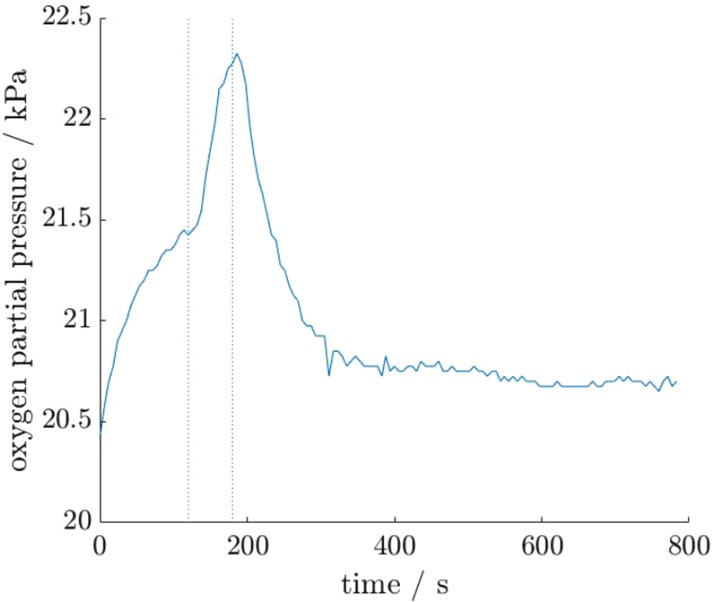
pO_2_ measured in gel phantom over time. Solution was exposed to ultrasound for 1 min during period marked out with dotted lines. Results shown are an average of n = 4

The oxygen concentrations in the microbubble suspensions were measured, both before and after exposure to ultrasound, using a blood parameter monitor. The results are shown in Fig. 1. There was a rapid increase in measured oxygen concentration when the samples were exposed to ultrasound. However, the increased pO_2_ was not maintained after the tubing was removed from the ultrasound bath. This indicates that oxygen was released from the microbubble upon cavitation, but did not remain in solution. This is consistent with the observation of gas pocket formation in the syringes mentioned above.

### 3.2 Magnetic resonance imaging (MRI)

#### 3.2.1 Microbubbles

**Fig. 2:**
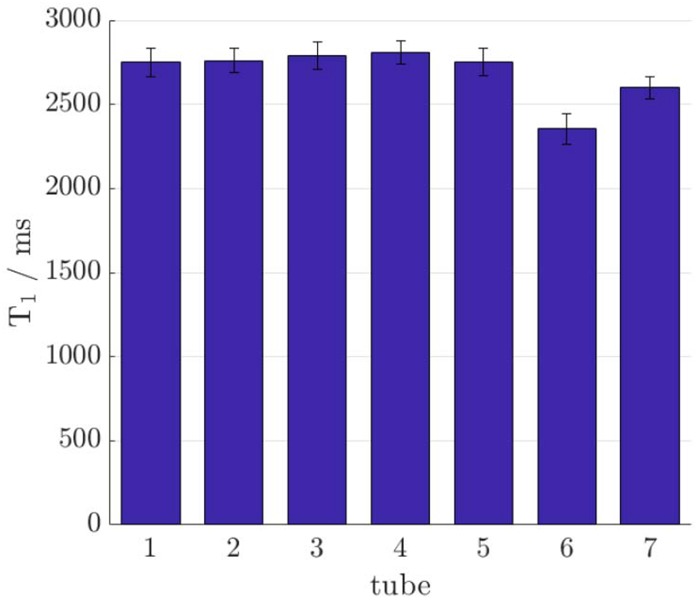
T1 measurements acquired with VFA. Tube numbers correspond to samples as follows: 1 – gel, 2 – (gel + 1 ml O_2_ microbubble), 3 – (gel + 0.25 ml O_2_ microbubble), 4 – (gel + 1 ml N_2_ microbubble), 5 – (gel + 0.25 ml N_2_ microbubble), 6 – oxygenated gel sparged for 120 s, 7 – oxygenated gel sparged for 20 s

The aim of the first experiment was to see if the addition of O_2_ microbubbles altered the T1 of the gel phantoms and to compare this with the effect of oxygen directly dissolved in the gel (Fig. 2). The T1 values for the O_2_ microbubble samples were not significantly different from those for the gel control when measured with either method. This supports the hypothesis that the gases are enclosed within the lipid shell of the bubbles, with minimal leakage into the external fluid. The tubes containing oxygenated gel showed the expected paramagnetic relaxation enhancement, with the lowest T1 corresponding to the longest sparging time. Although the T1 values obtained by the different methods were not the same, the relationships between the tubes were consistent. The infrared results had a much higher precision than those acquired with VFA, although this came at the cost of a much longer scanning time: 2 h and 8 min compared to just under 6 min for VFA. It is therefore difficult to say whether the discrepancies between the two methods (mainly an overestimate of T1 from VFA) are systematic or caused by temperature changes and gas leakage between scans. However, for this project, we were interested in the differences in T1 between samples, rather than the absolute values, and the VFA results were sufficiently accurate in this regard to continue using this method for the rest of the project.

**Fig. 3:**
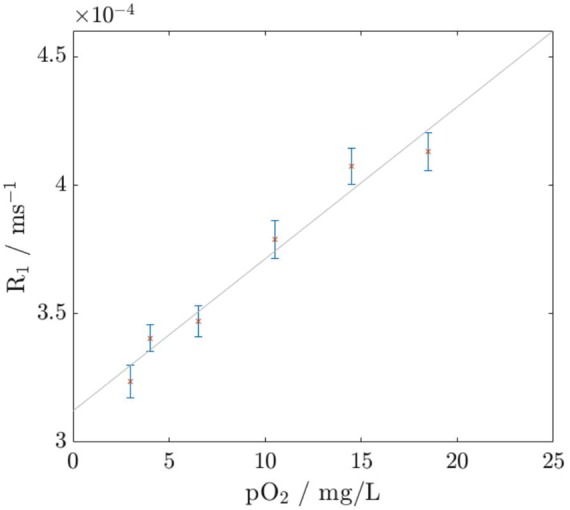
pO_2_, as measured with a bench-top dissolved oxygen probe, plotted against R1 measured with VFA from equivalent samples. Results were averaged over two runs and a linear fast Fourier transform (FFT) was calculated in Matlab. The linear regression and correlation coefficients are y = 0.3 + 0.006x, R^2^ = 0.96

For the next experiment, pO_2_ and T1 data were acquired from equivalent samples with differing concentrations of O_2_ and N_2_ gas. The nitrogenated gels were included to provide a wider range of concentrations, as dip-probe measurements indicated that sparging with nitrogen reduced the pO_2_ below baseline levels. The results are shown in Fig. 3. The procedure was repeated twice on different days and the T1–pO_2_ relationship was found to be consistent. This provided verification and quantification of the expected linear relationship between R1 = 1/T1 and pO_2_ in ultrasound gel, as well as generating results that could then be used to calibrate the oxygen released from the microbubbles in later experiments.

T1 was then measured in the O_2_ microbubble and N_2_ microbubble samples before and after submersion in the ultrasound bath to measure the change induced by the gas released from the bubbles. When the microbubble samples were exposed to ultrasound, the appearance of the solution changed from opaque to transparent and gas pockets could be seen forming in the ends of the tubes. This indicated, along with initial results showing no distinct change in T1 between scans, that the gas contents of the microbubble did not dissolve in the gel solution after the bubbles were ruptured. As described above the tubes were pressurised by depressing the plungers to increase gas solubility during subsequent scans. The results were averaged over two runs and normalised with respect to a gel control tube that underwent the same procedure to control for thermal effects.

**Fig. 4:**
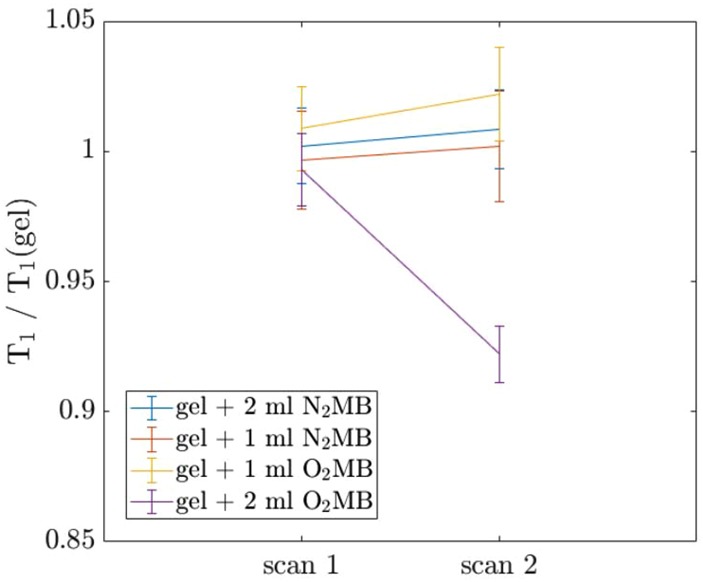
Change in T1 following ultrasound exposure in different microbubble suspensions

As shown in Fig. 4, the more concentrated O_2_ microbubble solution showed a significant decrease in T1 after the bubbles were ruptured (*p* = 0.02). None of the other samples showed statistically significant changes in T1. Using the calibration curve in Fig. 3, this corresponds to an increase in pO_2_ of around 4 mg/L (from 0.15 ml of microbubble suspension in a total sample volume of 0.5 ml). However, this T1 reduction was not observed in the phantoms with the lower O_2_ microbubble concentration. This indicates that there is likely to be a minimum concentration for which the technique is effective. The O_2_ microbubble and N_2_ microbubble samples were scanned 12 times over a period of 63 min. All samples showed approximately the same linear increase in T1 over the course of the experiment, which could be due to temperature increase induced by the switching magnetic field gradients. When the results were normalised with respect to the gel control, there were no significant trends observed in the T1 values with time. All samples remained within 5% of the gel value. This indicates that the bubbles were stable over this time period. The seemingly random fluctuations between consecutive scans illustrate the variability in this technique.

### 3.3 Limitations and further work

Temperature affects both T1 and gas solubility, so is clearly an important factor in these results. Attempts have been made to mitigate some of the effects of temperature by using controls, but it would be pertinent in future experiments to monitor the temperature of the samples and account for its effects quantitatively. In addition, a more realistic simulation of the effects in vivo could have been obtained by carrying out the experiments at 37°C. There are some results from this project which need to be investigated further if research is to be carried forward into pre-clinical models. Firstly, reduction in T1 could not be seen after the microbubble was ruptured unless the pressure of the samples was artificially increased. Quantitative measurements would need to be made of the pressures required to know whether blood pressure will be sufficient to see the same results. Repeating these experiments with blood phantoms would give a more accurate prediction for in vivo experiments.

## 4 Conclusion

In summary, oxygen loaded microbubbles are being developed as an agent for reducing tumour hypoxia in order to increase the effectiveness of cancer therapies. The effect of these oxygen carriers on the longitudinal relaxation times of gel phantoms was investigated, to test the viability of using VFA-MRI to monitor the outcomes of these treatments as well as their lifetime within the body. Intact microbubbles in gel phantoms did not induce a measurable change in T1, but with sufficiently high microbubble concentration, a reduction could be measured once the bubbles had been ruptured by ultrasound.
